# Supplementary material for: In vivo molecular imaging of chemokine receptor CXCR4 expression in patients with advanced multiple myeloma
Source: EMBO Mol Med. 2015 Mar 3;7(4):477–87. doi: 10.15252/emmm.201404698 (PMC4403048; doi:10.15252/emmm.201404698)
Supplement: Supplementary file 1 — Supplementary Figures [file emmm0007-0477-sd1.pdf]

Supplementary Information – manuscript: EMM-2014-04698-V3

Supplementary figure legends

Figure S1. Gating strategy: Plasma (myeloma) cells and leukocyte subpopulations for comparison. (**A**) Schematic diagram of the gating strategy applied to generate the data shown in Figure 1 and Figure S1B and S2. (**B**) Gating of Expression of CD184 in the CD38++/CD138+ population. CXCR4 (CD184-PE) expression levels were determined by comparing the fluorescence intensity of PE in CD184 positive plasma cells compared to the fluorescence intensity of the PE isotype control (mouse IgG 2a, κ) for plasma cells. Back-gating of CD184 positive plasma cells: Plasma cells were identified by color-coded back-gating of CD45/CD38, CD38/CD19 and typical FSC/SSC characteristics and then defined by CD138++CD38++. A first gate was set on the CD45 negative cells within the bone marrow mononuclear cells. Plasma cell gate: A second gate was set on CD45-/CD38++ cells and color back-gated on the first gate to ensure correct gating of plasma cells. Color back-gating was also used to ensure that plasma cells were CD38++/CD19- and with typical FSC/SSC properties. A representative analysis of one patient is shown.

Figure S2. Assessment of CXCR4 expression on bone marrow leukocyte populations. (**A**, **B**) A lymphocyte gate was set on CD45high cells. B-/T-cell gate: within the lymphocyte gate B-cells were identified by their characteristic expression of CD19 while T cells were defined as CD3+. CXCR4 (CD184-PE) levels were determined by comparing the fluorescence intensity of PE in CD184 positive B-/T-cells compared to the fluorescence intensity of the PE isotype control (mouse IgG 2a, κ) for lymphocytes. (**A**) Expression of CD184 in the CD19+ population. (**B**) Expression of CD184 in the CD3+ population. (**C**) Progenitor gate: a gate was first set on CD45low cells with low SSC characteristics. CD34+ progenitor gate: from the primary progenitor gate progenitors were further characterized by their expression of CD34 and expression of the CD33 antigen*. CXCR4 (CD184-PE) levels were determined by comparing the fluorescence intensity of PE in CD184 positive CD34+ progenitors compared to the fluorescence intensity of the PE isotype control (mouse IgG 2a, κ) for progenitors. (**D**) Gating of CD184 positive monocytes.

Monocyte gate: a gate was first set on CD45high cells with typical SSC characteristics. CD14+ monocyte gate: monocytes were further characterized by their expression of the CD14++ and the CD33+ antigen*. Expression of CD184 in the CD14++ population: CXCR4 (CD184-PE) levels were determined by comparing the fluorescence intensity of PE in CD184 positive monocytes compared to the fluorescence intensity of the PE isotype control (mouse IgG 2a, κ) for monocytes. (A-D) Analysis of the same representative patient as in Fig. S1 is shown. *Of note: CD33 staining in PECy7 is relatively weak. Normally we employ CD33 in PE but this was not possible since CD184 was used in PE.

Figure S3. AMD3100 blocks [^{68}Ga]Pentixafor uptake in human MM cell lines in vivo and in vitro. (**A**) NOD.CB17-*Prkdc*^{scid}/NCrHsd mice (n=4) bearing OPM-2 and MM.1S xenografts were coinjected with AMD3100 or not coinjected before undergoing [^{68}Ga]Pentixafor-PET. Shown is the mean tumor-to-background (TBR) ± SEM. *p=0.0008 (MM1S); *p=0.0003 (OPM-2); unpaired Student's t-test. (**B**) In vitro uptake studies in the indicated MM cell lines. Cells were pretreated for 30 minutes with AMD3100 100µM before [^{68}Ga]Pentixafor incubation. The data show the uptake of the radionuclide. One experiment was performed (technical triplicate).

Figure S4. [^{68}Ga]Pentixafor-PET negative images. Maximum intensity projections (MIP) of [^{68}Ga]Pentixafor (**A**) and [^{18}F]FDG PET/CT (**B**) of a patient with histologically proven multiple myeloma. Trans-axial views of the upper abdomen (**C**) demonstrate no increased [^{68}Ga]Pentixafor uptake (upper row) and no increased [^{18}F]FDG uptake compared to physiological background uptake (lower row) in projection of the osteolytic lesion (yellow arrows).

Figure S5. [^{68}Ga]Pentixafor and [^{18}F]FDG PET/CT. Maximum intensity projections (MIP) of [^{68}Ga]Pentixafor (**A**) and [^{18}F]FDG PET/CT (**B**) of a 74 year old male with histologically proven multiple myeloma. Trans-axial views of the head (**C**), thorax (**D**), lower thorax/abdomen (**E**) and pelvis (**F**) demonstrate the higher sensitivity of [^{68}Ga]Pentixafor (left images) compared to [^{18}F]FDG (right images) in this patient. Exemplary shown are several lesions (yellow arrows).

Figure S6. [^{68}Ga]Pentixafor and [^{18}F]FDG PET/CT uptake values. (**A**) Display of maximum standard uptake values (SUVmax) for [^{68}Ga]Pentixafor and [^{18}F]FDG PET/CT for all lesions (n=32) measured. Box-Whisker plots of SUVmax are shown; p=0.018 (Wilcoxon Signed Rank test). (**B**) Display of tracer uptake values of lesions separated into [^{18}F]FDG positive and [^{68}Ga]Pentixafor negative (n=6; left), [^{18}F]FDG negative and [^{68}Ga]Pentixafor positive (n=9; middle), and lesions visually positive for [^{18}F]FDG and positive for [^{68}Ga]Pentixafor (n=17; right). Box-Whisker plots of SUVmax are shown.

Figure S7. [^{68}Ga]Pentixafor PET does not affect blood counts or mobilize CD34^{+} cells into the peripheral blood. Blood samples were drawn 1 hour before (-1), 1 hour (+1), 24 hours (+24) and 7 days after [^{68}Ga]Pentixafor PET imaging (day 7). (**A**) CD34^{+} cells given as % of CD45^{+} cells. (**B**) White blood cell count (WBC). (**C**) Hemoglobin. (**D**) Platelet count. None of the differences in (**A**)-(**D**) are statistically significant (ANOVA).

Figure S1

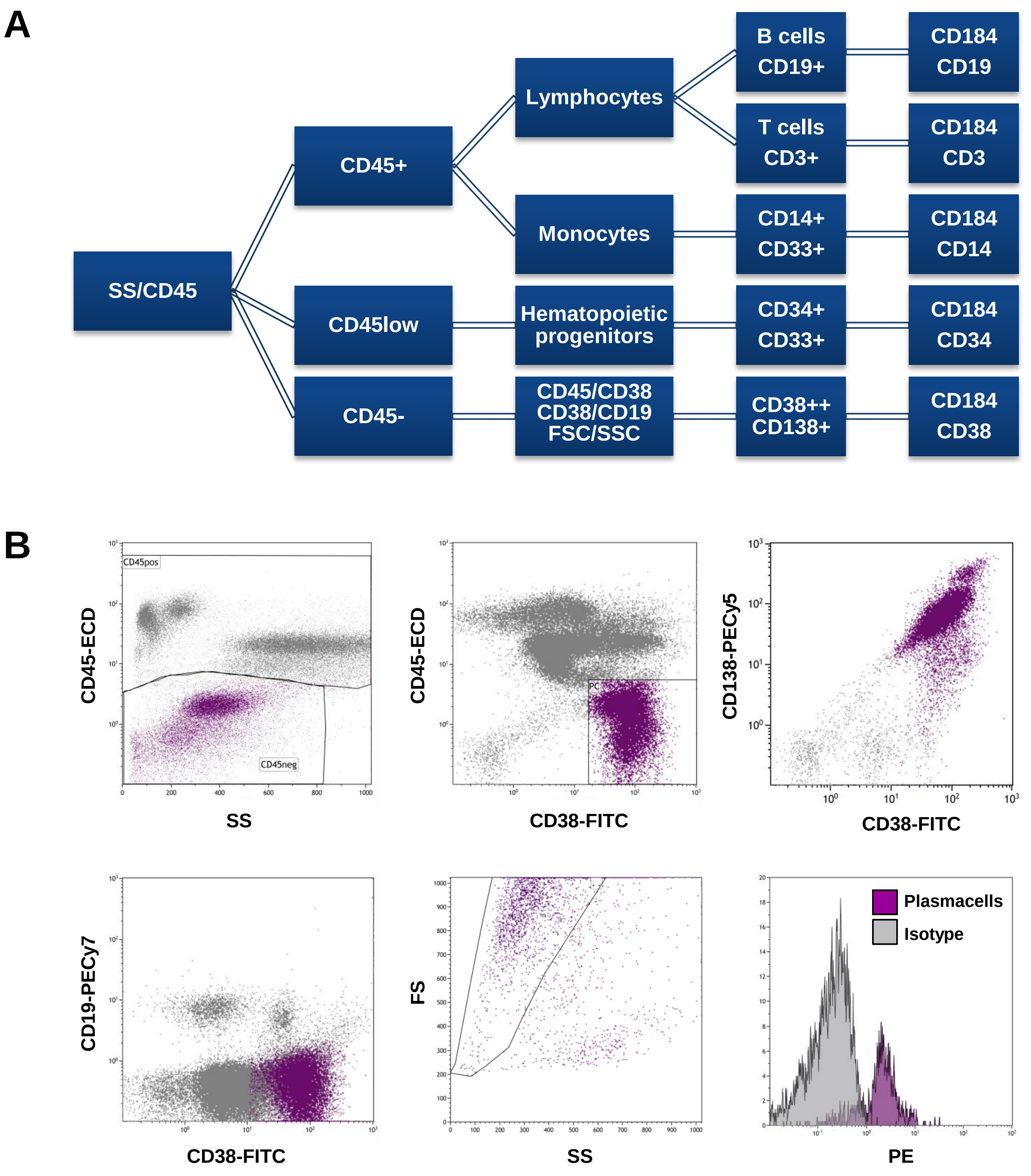
A
SS/CD45
CD45+
CD45low
CD45-
Lymphocytes
Monocytes
Hematopoietic progenitors
CD45/CD38
CD38/CD19
FSC/SSC
B cells
CD19+
T cells
CD3+
CD14+
CD33+
CD34+
CD33+
CD38++
CD138+
CD184
CD19
CD184
CD3
CD184
CD14
CD184
CD34
CD184
CD38
B
CD45pos
CD45neg
CD45-ECD
SS
PC
CD38-FITC
CD138-PECy5
CD19-PECy7
FS
Plasmacells
Isotype
PE

Figure S2

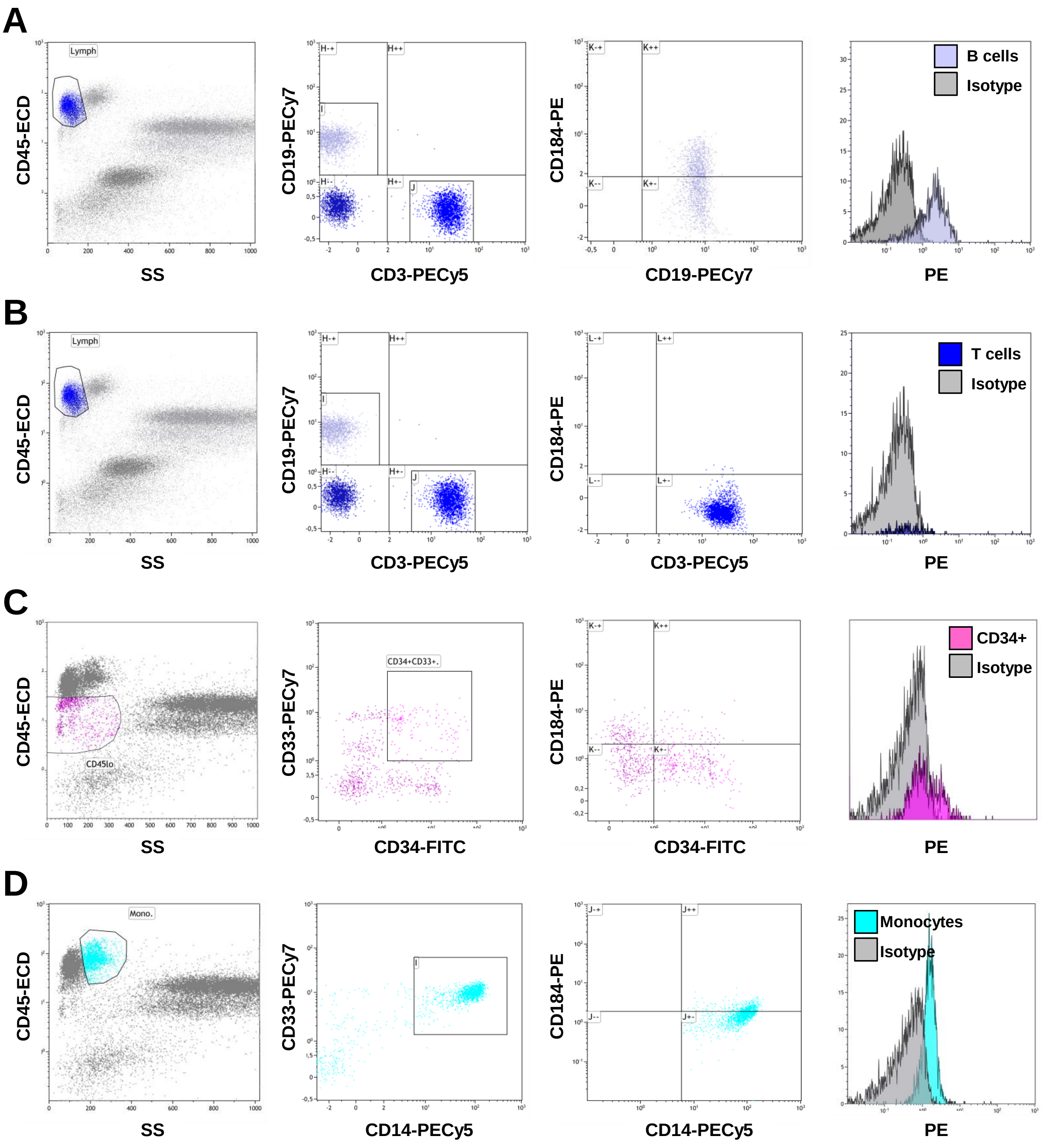

A
Lymph
CD45-ECD
SS
CD19-PECy7
CD3-PECy5
CD184-PE
CD19-PECy7
B cells
Isotype
PE
B
Lymph
CD45-ECD
SS
CD19-PECy7
CD3-PECy5
CD184-PE
CD3-PECy5
T cells
Isotype
PE
C
CD45lo
CD45-ECD
SS
CD34+CD33+.
CD33-PECy7
CD34-FITC
CD184-PE
CD34-FITC
CD34+
Isotype
PE
D
Mono.
CD45-ECD
SS
CD33-PECy7
CD14-PECy5
CD184-PE
CD14-PECy5
Monocytes
Isotype
PE

Figure S3

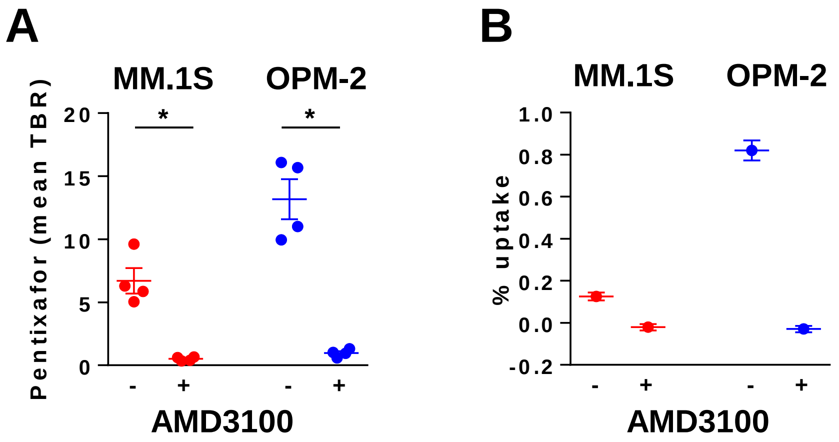
A
MM.1S
OPM-2
Pentixafor (mean TBR)
20
15
10
5
0
*
*
-
+
-
+
AMD3100
B
MM.1S
OPM-2
% uptake
1.0
0.8
0.6
0.4
0.2
0.0
-0.2
-
+
-
+
AMD3100

Figure S4

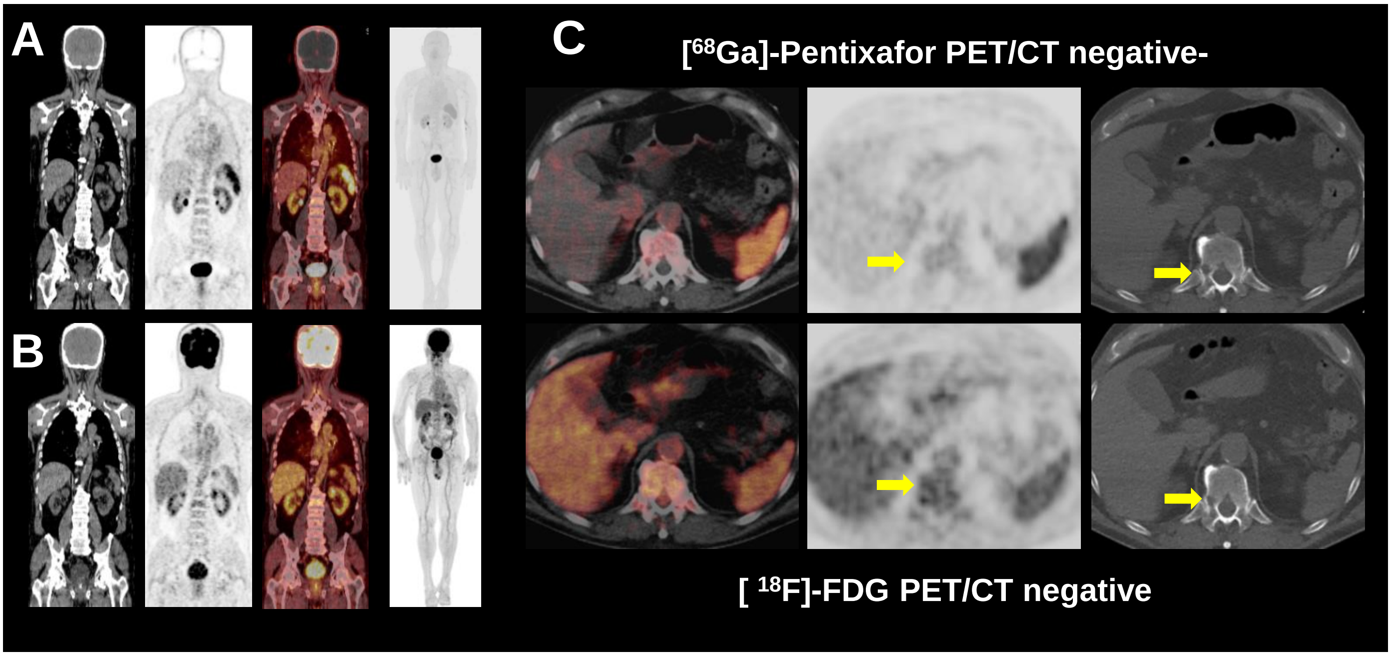

Figure S5

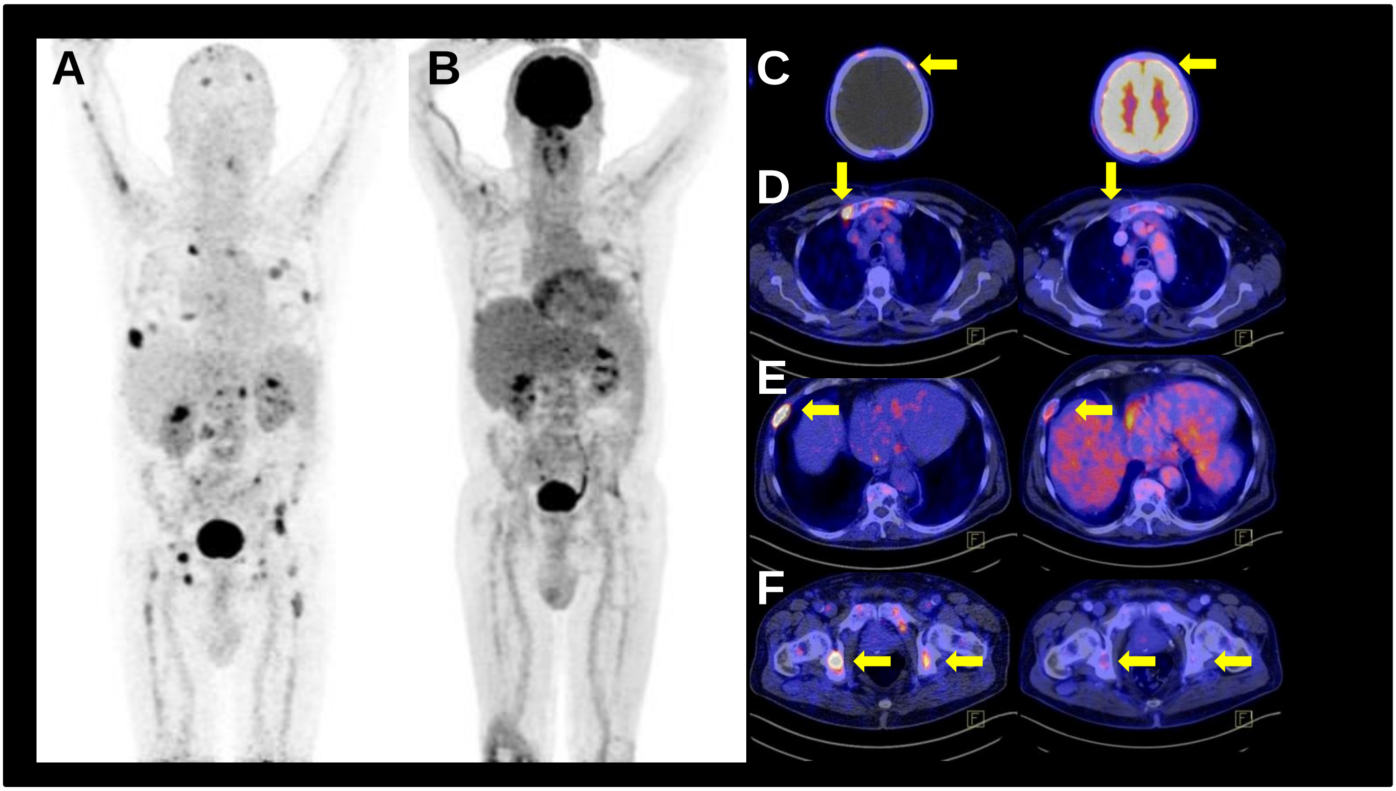

Figure S6

A

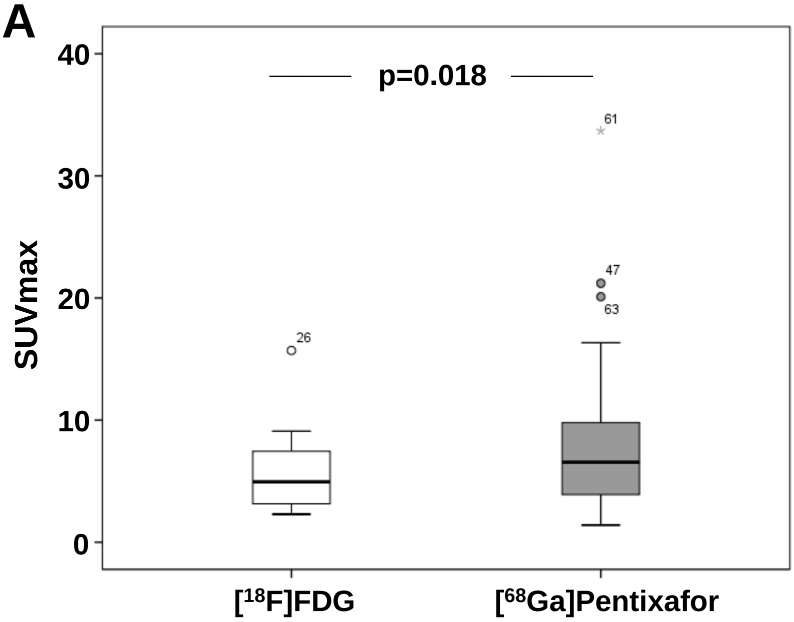

p=0.018
40
30
20
10
0
SUVmax
61
47
63
26
[18F]FDG
[68Ga]Pentixafor

B

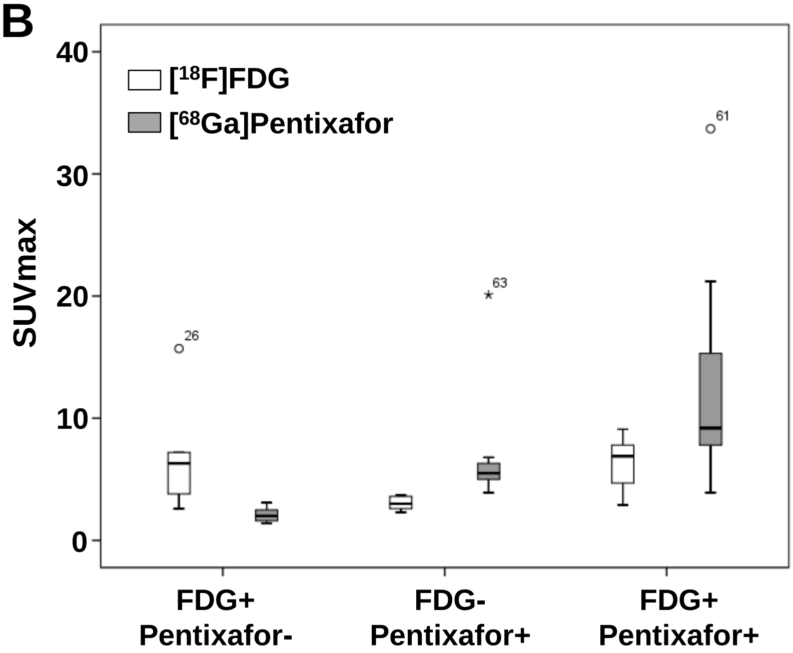

[18F]FDG
[68Ga]Pentixafor
40
30
20
10
0
SUVmax
61
63
26
FDG+
Pentixafor-
FDG-
Pentixafor+
FDG+
Pentixafor+

Figure S7

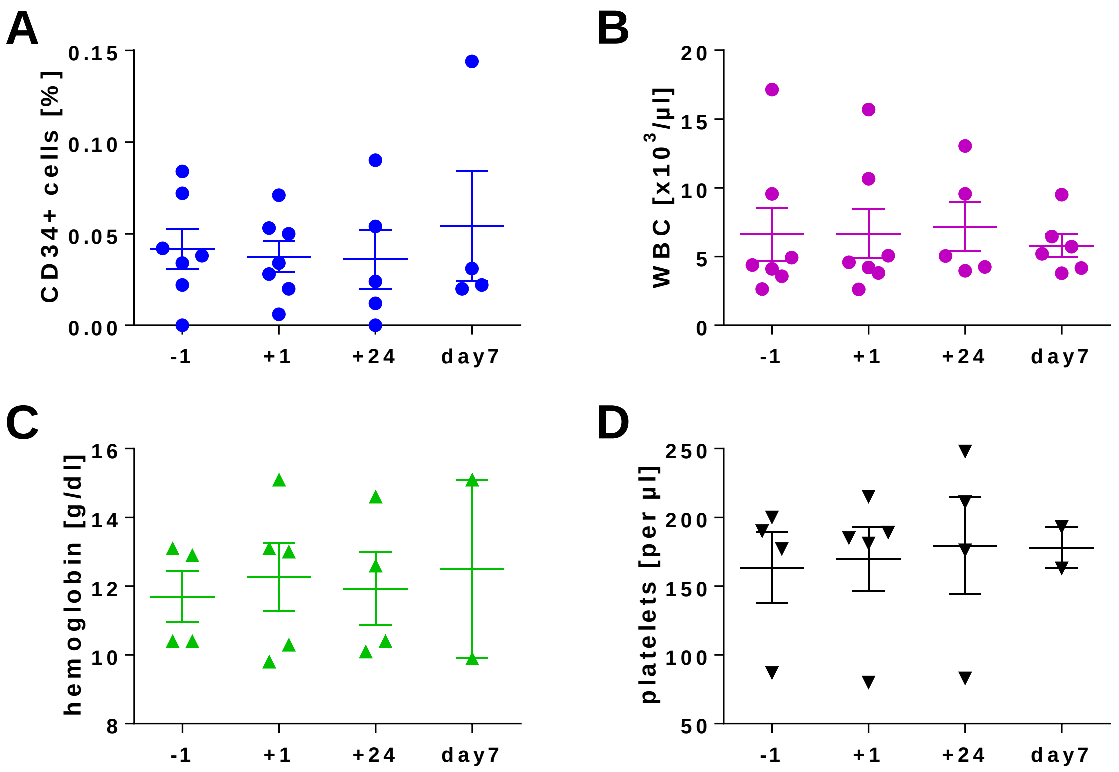

A
CD34+ cells [%]
0.15
0.10
0.05
0.00
-1
+1
+24
day7
B
WBC [x10^3/µl]
20
15
10
5
0
-1
+1
+24
day7
C
hemoglobin [g/dl]
16
14
12
10
8
-1
+1
+24
day7
D
platelets [per µl]
250
200
150
100
50
-1
+1
+24
day7
