# Supplementary figures and images for: In vivo molecular imaging of chemokine receptor CXCR4 expression in patients with advanced multiple myeloma
Source: EMBO Mol Med. 2015 Mar 3;7(4):477–87. doi: 10.15252/emmm.201404698 (PMC4403048; doi:10.15252/emmm.201404698)

Figure 2B

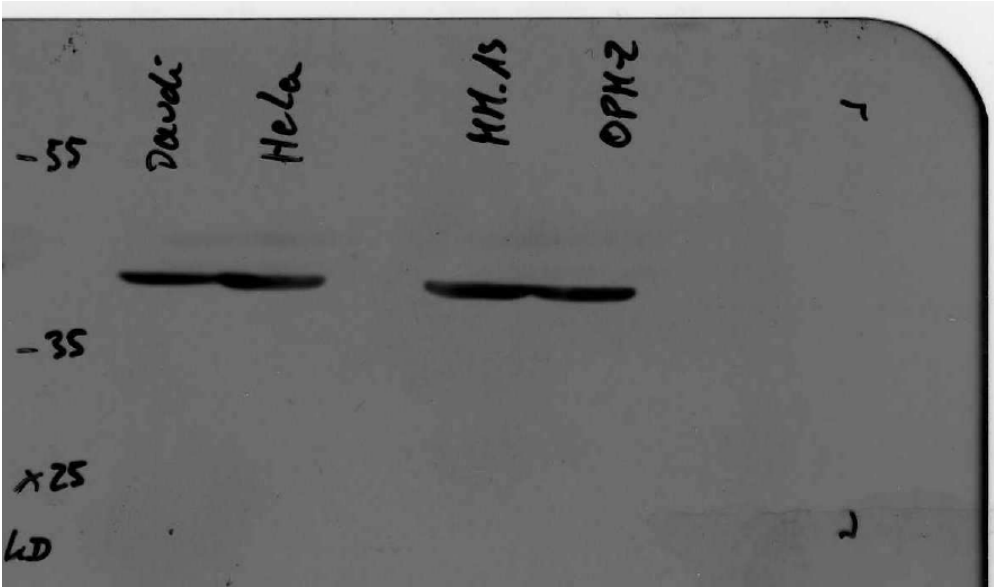

Actin

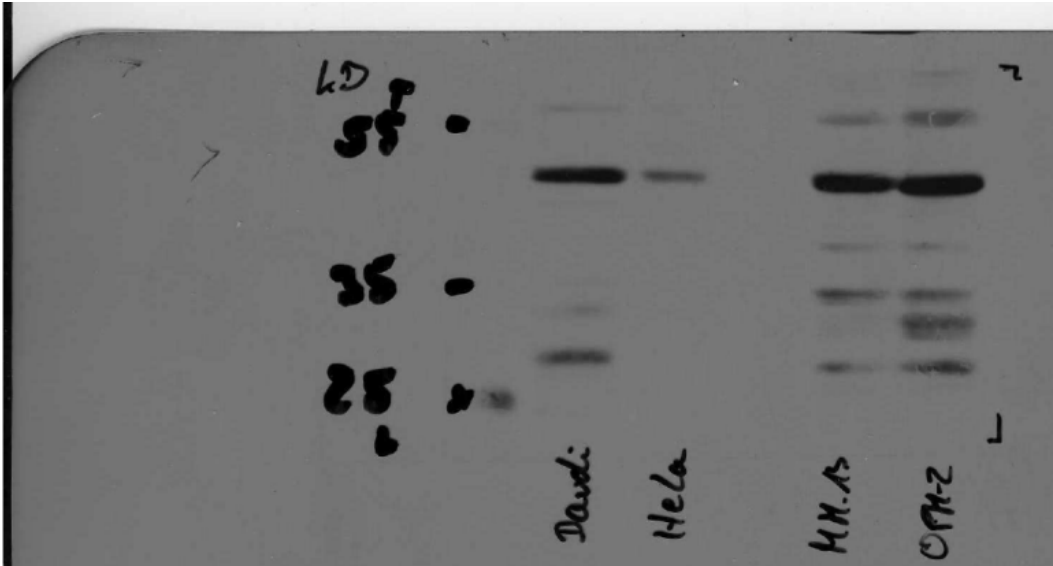

Cxcr4

Supplement: Supplementary file 3 — Source data for Figure 2 [file emmm0007-0477-sd3.pdf]
